# 

**Published:** 1903-03

**Authors:** 


### CONTENTS.

ORIGINAL ARTICLES:
Foot-and-Mouth Disease in Massachusetts. By AUSTIN PETERS, M.R.C.V.S. . . . 133
Observations on Country Practice. By S. H. BAUMAN, D.V.S. . . . . . . . 144
Compressed Air in the Treatment of Ulcers and Fistulas. By ARNOTT A. ADAMSON, M.D.V. 146
Vitality and Immunity. By CARL SCHULIN, M.D. . . . . . . . . . . 147
Hemorrhagic Septicæmia. By M. H. REYNOLDS, M.D., V.M. . . . . . . . . 156
ABSTRACTS FROM FOREIGN JOURNALS . . . . . . . . . . . . . 162
REPORTS OF CASES:
Three Anomalies I Have Met with in Castration. By J. R. SANDERS, M.D.C. . . . 164
The Serum Treatment in Purpura Hemorrhagica. By J. H. MCLEOD, D.V.S. . . . 166
Fracture of the Ribs in a Horse. By WILLIAM DRINKWATER, V.S. . . . . . . . 168
Three Cases Showing the Use of Oil of Turpentine in the Treatment of Atrophy of the Shoulder Muscles. By W. A. HECK, V.S. . . . . . . . . . . . 169
EDITORIALS:
Amendments Before the Michigan Legislature . . . . . . . . . . 172
Bureau of Animal Industry . . . . . . . . . . . . 172
The Passing Shadow . . . . . . . . . . . . . . 172
BUREAU OF ANIMAL INDUSTRY . . . . . . . . . . . . . 173
NOTES . . . . . . . . . . . . . . . . . . 175
SOCIETY PROCEEDINGS . . . . . . . . . . . . . . 178
PERSONALS . . . . . . . . . . . . . . . . 199

